# Uremic frost: a historical note

**DOI:** 10.1093/ckj/sfaf368

**Published:** 2025-11-28

**Authors:** Søren H Elsborg, Rikke Nørregaard, Henricus A M Mutsaers

**Affiliations:** Department of Clinical Medicine, Aarhus University, Aarhus, Denmark; Department of Clinical Medicine, Aarhus University, Aarhus, Denmark; Department of Renal Medicine, Aarhus University Hospital, Aarhus, Denmark; Department of Clinical Medicine, Aarhus University, Aarhus, Denmark

To the Editor,

Uremic frost is a rare but striking dermatologic manifestation of advanced kidney failure, characterized by the formation of urea crystals on the skin after sweat evaporates. It typically occurs when blood urea nitrogen exceeds 200 mg per deciliter [1–4]. While now uncommon, in the era before dialysis it served as an important marker of severe renal dysfunction. Modern sources often state that uremic frost was first described by the Danish pediatrician Harald Hirschsprung in 1865. However, careful examination of the original texts suggests that this is not entirely accurate.

In his 1865 article ‘Urinstof paa Huden og Uræmi’ in Ugeskrift for Læger, Hirschsprung reflected on a case he had published 7 years earlier: ‘In the spring of 1858, […] I reported a case of cancer of the urinary bladder, which ended in uremia, and in which urea appeared on the skin of the face towards the end of life, I could not show any perfectly analogous case; but I could only refer to a recently published treatise by Draschke’. Here, he is referring to the work of the Austrian internist and epidemiologist Dr Anton Drasche, who, during the cholera epidemic in Vienna (1855), observed 12 patients (out of 800) with ‘urea coating’ and little to no urine excretion. In his 1856 publication, ‘Über den Harnstoff-Beschlag der Haut und Schleimhäute im Cholera-Typhoid’ in Zeitschrift der k. k. Gesellschaft der Ärzte zu Wien, Drasche noted that ‘an essential component of the urine can crystallize freely on the surface of the body’, and that ‘the first crystalline deposit usually appears on the eyebrows, then the temples, the nostrils, forehead, upper lip, the hairy part of the skull, […] in the above-mentioned order’. More importantly, he analyzed the crystals and concluded ‘that the crystalline compound […] is real and true urea’. Thus, to the best of our knowledge, Drasche’s account appears to be the earliest verifiable description of what we now call uremic frost.

Nevertheless, Hirschsprung’s contribution remained crucial to our current understanding of uremic frost. While Drasche was the first to observe and chemically confirm urea crystals on the skin of cholera patients with anuria, Hirschsprung, through careful literature review and his own clinical observations, was the first to confidently link these deposits to kidney failure.

Revisiting these historical sources reminds us how careful clinical observation can shape medical understanding and that a keen eye remains a cornerstone of medical practice.
